# Resistant and refractory migraine: clinical presentation, pathophysiology, and management

**DOI:** 10.1016/j.ebiom.2023.104943

**Published:** 2023-12-23

**Authors:** Raffaele Ornello, Anna P. Andreou, Eleonora De Matteis, Tim P. Jürgens, Mia T. Minen, Simona Sacco

**Affiliations:** a Department of Biotechnological and Applied Clinical Sciences, University of L'Aquila, L'Aquila, Italy; b Headache Research-Wolfson CARD, Institute of Psychiatry, Psychology and Neuroscience, King's College London, London, UK; c Headache Centre, Guy's and St Thomas' NHS Foundation Trust, London, UK; d Headache Center North-East, Department of Neurology, University Medical Center Rostock, Rostock, Germany; e Department of Neurology, KMG Hospital Güstrow, Güstrow, Germany; f Departments of Neurology and Population Health, NYU Langone Health, New York, USA

**Keywords:** Resistant migraine, Refractory migraine, CGRP, Non-pharmacological treatments, Migraine-related disability

## Abstract

Migraine is a leading cause of disability worldwide. A minority of individuals with migraine develop resistant or refractory conditions characterised by ≥ 8 monthly days of debilitating headaches and inadequate response, intolerance, or contraindication to ≥3 or all preventive drug classes, respectively. Resistant and refractory migraine are emerging clinical definitions stemming from better knowledge of the pathophysiology of migraine and from the advent of migraine-specific preventive treatments. Resistant migraine mostly results from drug failures, while refractory migraine has complex and still unknown mechanisms that impair the efficacy of preventive treatments. Individuals with resistant migraine can be treated with migraine-specific preventive drugs. The management of refractory migraine is challenging and often unsuccessful, being based on combinations of different drugs and non-pharmacological treatment. Future research should aim to identify individuals at risk of developing treatment failures, prevent the condition, investigate the mechanisms of refractoriness to treatments, and find effective treatment strategies.

## Introduction

Migraine is the second cause of disability worldwide.^1^ A small, yet undefined proportion of individuals with migraine experiences disabling attacks that acute and preventive strategies fail to control. These individuals have resistant or refractory migraine depending on the number of unsuccessful preventive treatments. Reviewing the clinical scenarios, mechanisms, and therapeutic strategies specific to resistant and refractory migraine is especially timely given the availability of new drugs for individuals with resistant migraine and advancements in the migraine field that might promote the understanding, prevention, and treatment of refractory migraine.

### Search strategy and selection criteria

We selected references by searching PubMed, the Cochrane library, MEDLINE, and Embase and for manuscripts published in English between Jan 1, 2013, and June 31st, 2023. We used the term “migraine” combined with the terms “resistant”, “refractory”, “prevention”, “failure”, “disabling”, “debilitating”, “neurophysiology”, “neuroimaging”, “magnetic resonance imaging”, “electroencephalogram”, “diet”, “exercise”, “sleep”, “neuromodulation”, and “behavioural therapy”. We included original data, meta-analyses, consensus statements, guidelines, reviews, comments, and opinions dealing with difficult-to-treat migraine, resistant or refractory migraine. The final reference list was based on the relevance to the topic. We prioritised publications within the last 5 years.

### Definitions

**Fig. 1:**
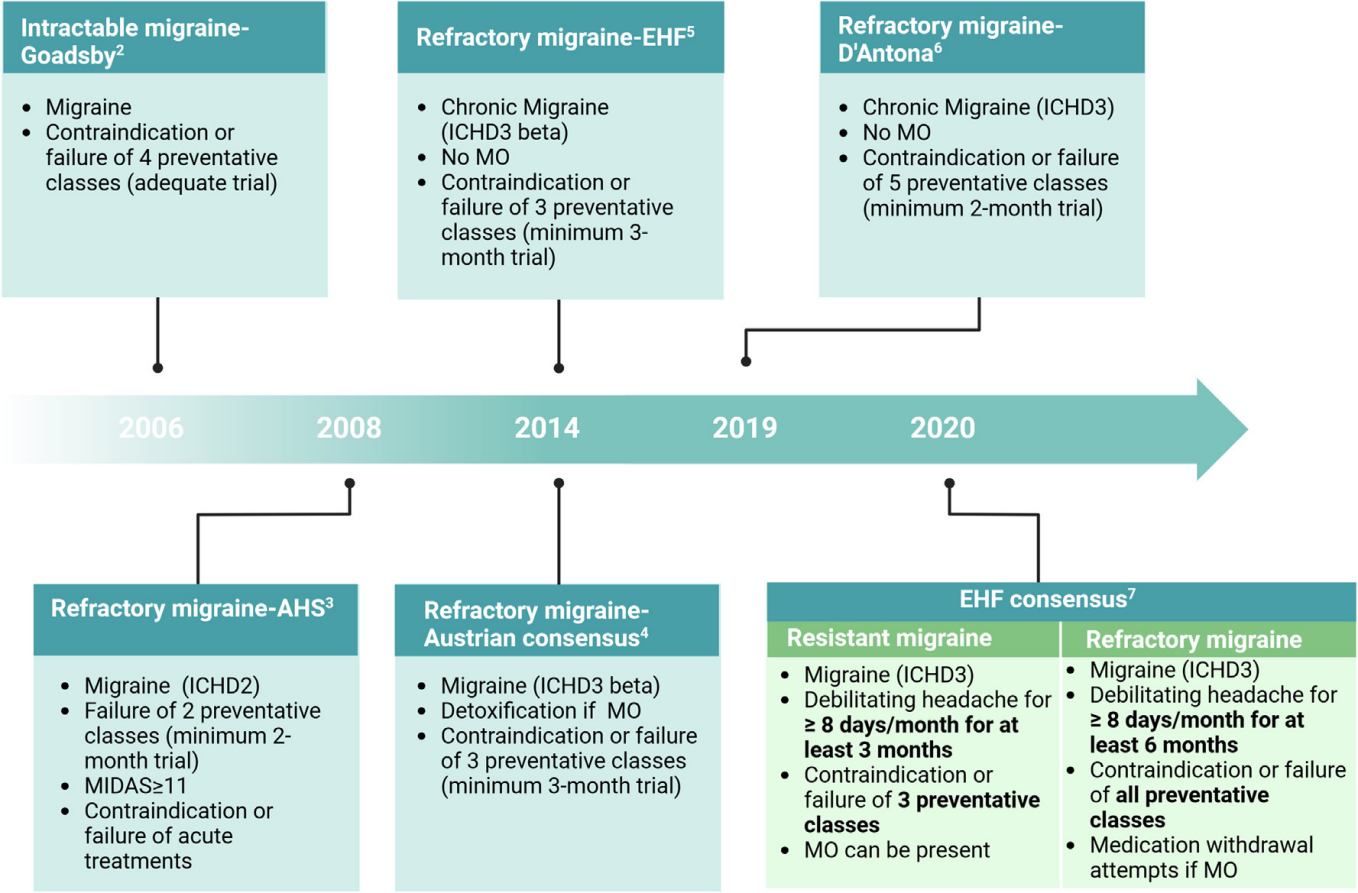
**Definitions of difficult-to-treat migraine proposed through the years**. Definitions of refractory migraine changed over time. The most recent European Headache Federation consensus distinguishes resistant and refractory migraine according to the number of contraindication or failure of prior preventative classes and of consecutive months with at least 8 debilitating headaches. Treatment failure is declared after an adequate attempt (i.e., adequate dose and duration: 2 months for oral preventatives, 3 months for monoclonal antibodies targeting the calcitonin gene-related peptide, 6 months for onabotulinumtoxinA). An attack is debilitating when causes serious impairment to daily activities despite the use of an adequate dose of symptomatic medication. Abbreviations: *AHS*: American Headache Society; *EHF*: European Headache Federation; *ICHD*: International Classification of Headache Disorders; *MO:* medication overuse; *MIDAS*: Migraine Disability Assessment.

At variance with previous definitions of difficult-to-treat migraine^2^, ^3^, ^4^, ^5^, ^6^ (Fig. 1), which defined a single clinical entity, the European Headache Federation (EHF) definitions of 2020 distinguished resistant from refractory migraine, based on the failure of ≥3 classes of preventive drugs for resistant migraine and failure of all classes of preventive drugs for refractory migraine.^7^ Resistant migraine can evolve into refractory migraine as more preventive treatments fail.

**Fig. 2:**
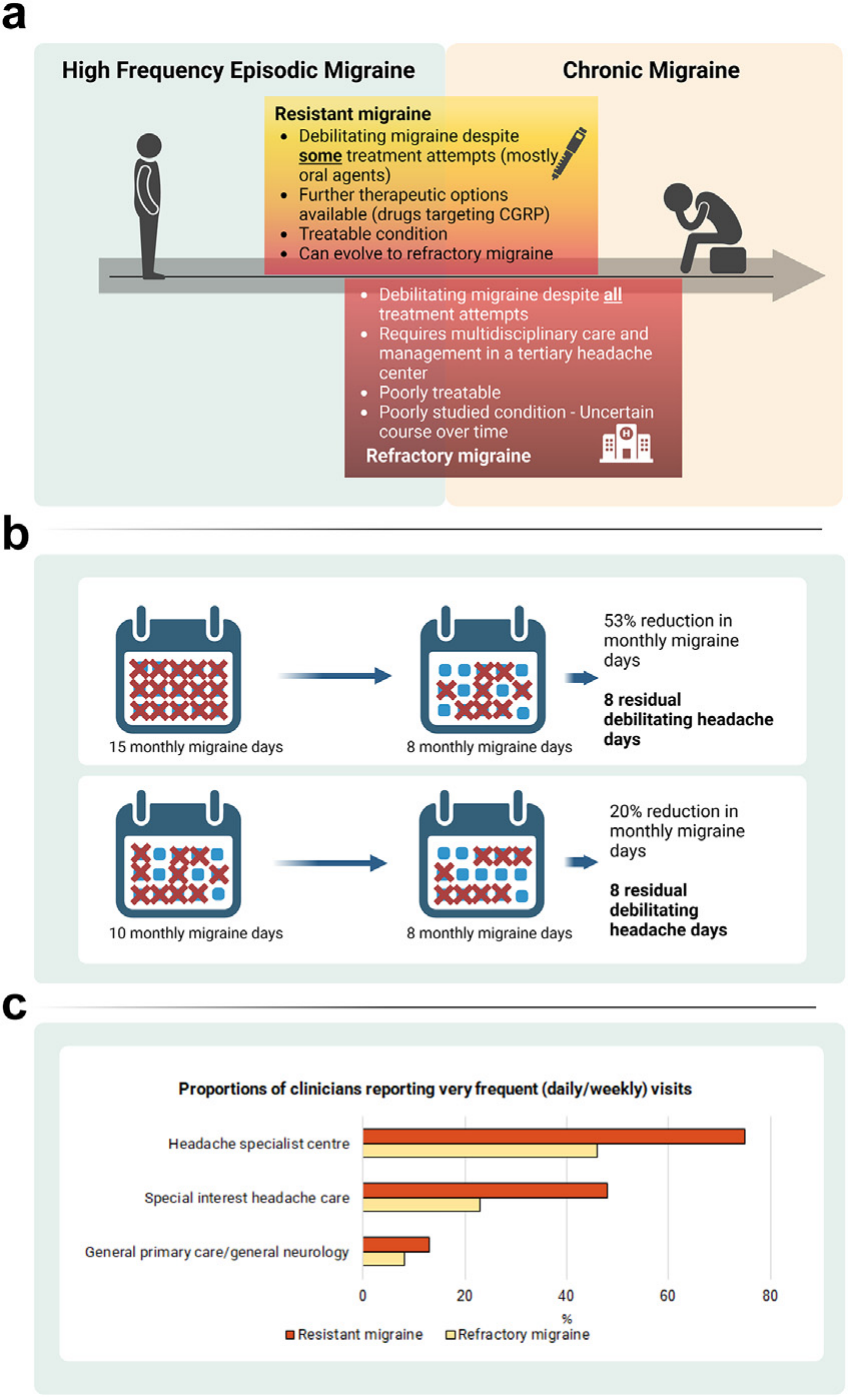
**a. Characteristics of resistant and refractory migraine. b. The importance of residual monthly migraine days. c. Prevalence of resistant and refractory migraine in headache centres.a**. Individuals with high frequency episodic or chronic migraine might meet definition of resistant and refractory migraine when reporting a high disability burden and failures of different classes of preventatives. Most individuals with refractory migraine have chronic migraine. Resistant migraine still has therapeutic options to ameliorate the condition, but may also eventually evolve to refractory migraine. **b**. The residual burden of migraine after prevention could be significant even if the preventative has a high relative efficacy; in those subjects, the residual burden of migraine could be similar to that of individuals with low response to preventive treatments. **c**. Proportion of clinicians reporting frequent (weekly to daily) visits to individuals with resistant or refractory migraine. Data from the Burden and Attitude towards Resistant and Refractory migraine (BARR) survey.^8^

Treatment failure is defined either as a lack of efficacy after trying a drug class for an adequate duration at an adequate dose, intolerable adverse events, or contraindication. Contraindication is included among the possible causes of medication failure as it forces the individual not to take the medication and is, therefore, not different from intolerance. The available classes, doses, and durations of preventive treatments were coded in the 2020 EHF definition together with contraindications. Of note, medication classes could be trialled in any order, without distinction into first or second lines. In both resistant and refractory migraine, individuals have ≥8 monthly days of debilitating headache for at least 3 and 6 months, respectively.^7^ The presence of debilitating headache days implies that treatment of acute attacks is also unsuccessful in individuals with resistant or refractory migraine. Individuals with either high-frequency episodic migraine (EM) or chronic migraine (CM) can have resistant or refractory migraine (Fig. 2a). The presence of medication overuse (i.e., overuse of symptomatic treatments for migraine) does not exclude any of the diagnoses, but documented acute drug withdrawal attempts are mandatory to diagnose refractory migraine.^7^

Notably, the EHF definitions consider the residual number of monthly headache days, irrespective of the reduction in monthly migraine or headache days obtained with preventive treatments. Individuals with ≥30% or ≥50% reduction in monthly migraine or headache days experience in some cases substantial residual migraine days (Fig. 2b).

## Epidemiology

Given the recency of definitions of resistant and refractory migraine, the epidemiology of those conditions has not been systematically explored. Resistant and refractory migraine are expected to be rare in the general population, but frequent in headache centres. An international survey showed that individuals with resistant migraine were seen at least weekly by 11/83 (13%) of physicians in general primary care or neurology, 34/71 (48%) of those with a special interest in headache care, and 92/123 (75%) of physicians in headache specialist centres; the corresponding proportions for refractory migraine were 7/83 (8%), 16/71 (23%) and 57/123 (46%) respectively (Fig. 2c).^8^

## Clinical features

Resistant and refractory migraine can be present in high-frequency EM or CM and in individuals with and without medication overuse. Resistant migraine might become treatable when a more effective or tolerable preventive treatment is started. In clinical practice, treatment consisting of oral agents not specific to migraine had failed in most of the individuals with resistant migraine. In fact, reimbursement policies restrict the use of monoclonal antibodies (mAbs) targeting the calcitonin gene-related peptide (CGRP) pathway to individuals with prior treatment failures and the use of onabotulinumtoxinA is limited to CM. Resistant migraine can also progress to refractory migraine in case of insufficient response to escalation treatments. Many individuals with resistant or refractory migraine become frustrated in their long journey of misdiagnoses, unnecessary investigations, and ineffective treatments.^9^

**Fig. 3:**
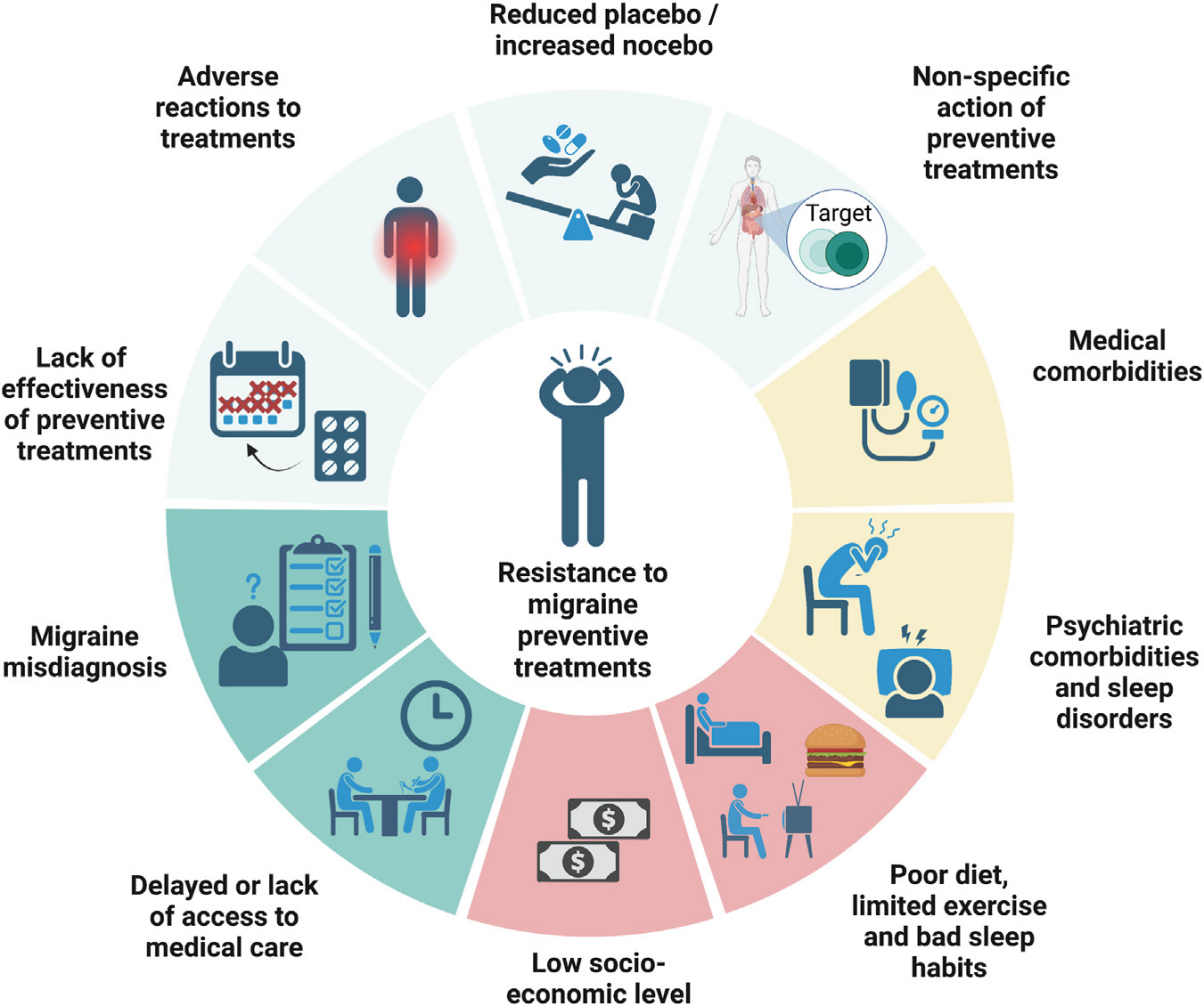
**Factors leading to or promoting resistance and refractoriness to preventative migraine drugs**. Main factors that can play a role in the onset and progression of resistant and refractory migraine. Those factors can be related to the drug (light green), individual comorbidities (light yellow), lifestyle (light red) and the disease (dark green) such as misdiagnosis of migraine—i.e., correct diagnosis many years from onset—and delayed care, which can also contribute to resistance to treatments. See sections 4 and 5 of the text for references.

Fig. 3 reports some factors that might predispose to treatment refractoriness. Those factors are not directly linked to resistance to preventive treatments; nevertheless, they are risk factors for migraine worsening and chronification, which is a form of migraine progression. Pain-related comorbidities such as fibromyalgia, irritable bowel disease, and complex regional pain syndrome are associated with central sensitisation to pain.^10^ Studies are needed to investigate how these pain-related comorbidities affect resistance to treatments. Psychiatric comorbidities could favour progression to CM through hyperactivity of the limbic system, which is implicated in the regulation of both behaviour and pain perception.^11^ Stress can decrease response to acute treatments,^12^ thus, leading to medication overuse. Pain catastrophising, feelings of helplessness, and ruminative thinking are also more pronounced in individuals with migraine—and mostly CM—compared with those without migraine,^13^ which might favour nocebo effect. Head trauma has also been associated with the transformation of EM into CM with medication overuse.^14^ Low socioeconomic status and income, and poor social support could contribute to migraine chronification^15^ and thus to migraine worsening which might confer resistance to treatments.

## Basis for resistance and refractoriness

Treatment failure in migraine can derive from lack of efficacy, poor tolerability, and loss of treatment effect (Fig. 3). Lack of efficacy and tolerability of migraine preventive drugs can be related to the lack of specificity of oral treatments that have unclear mechanisms of action. This issue is at least in part overcome by recent migraine-specific preventive agents such as CGRP-mAbs and gepants. Specifically, CGRP-mAbs inhibit CGRP signalling, in the case of erenumab, by binding to the extracellular domain of the CGRP receptor (CGRPr) or, in the cases of fremanezumab, galcanezumab, and eptinezumab, by preventing the interaction between CGRP and its receptor.^16^ Gepants peripherally bind to the extracellular domain of CGRPr and are subsequently internalised along with CGRPr blocking its endosomal residual action.^17^

Regarding efficacy, the estimated therapeutic gain of oral preventatives compared with placebo ranges from −0.4 to −1.5 monthly headache days,^18^ whereas CGRP-mAbs led to a reduction of 2–3 monthly migraine days (MMDs) compared with placebo.^19^ Placebo response was also higher for injectable CGRP-mAbs compared with oral drugs, resulting in a overall greater benefit in clinical practice. However, even individuals with a high response to preventive treatments might still have a remarkable residual disease burden. Indeed, the ESTEEMen study reported that among individuals having 50–74% reduction in MMDs after erenumab treatment, approximately one quarter still had 8-14 residual MMDs.^20^

Regarding tolerability, individuals with migraine have a high risk for adverse effects to preventive drugs. One study showed that topiramate led to more adverse events in individuals treated for migraine than in those treated for epilepsy.^21^ Nocebo effect is a potential explanation for the excess of adverse events,^22^ possibly triggered by central sensitisation^23^ or to the low expectancy of benefit from non-specific drugs. A combination of poor efficacy and poor tolerability determines poor adherence to preventive treatments, which is estimated as 25% at six months and 14% at 12 months for oral drugs.^24^

A third reason for treatment failure—together with poor efficacy and tolerability—is loss of treatment effect over time, a phenomenon observed for oral migraine preventatives and yet understudied. Loss of treatment effect can derive from treatment-dependent and treatment-independent mechanisms. Treatment-dependent mechanisms include pharmacokinetic and pharmacodynamic tolerance, by which the mechanisms leading to migraine adapt themselves and escape the action of drugs, and drug-induced disease progression, by which the repeated administration of drugs can trigger migraine worsening. Treatment-independent mechanisms include an initial placebo effect that is lost over time, spontaneous fluctuations in migraine course that lead to disease progression despite the use of preventive drugs, inaccurate recall of treatment effects, and drug delivery problems resulting in poor quality of the drugs.^25^

## Pathophysiology

Resistant migraine may be interpreted as a pure failure of migraine preventive treatments, while refractory migraine likely implies changes in sensory processing and neurotransmitters that are still unknown. The pathophysiological study of resistant and refractory migraine currently lacks direct evidence. Studies in CM may shed some light into the mechanisms that drive refractoriness, assuming that high frequency and resistance to treatments are both signs of migraine progression. Notably, whole-genome sequencing found no genetic variants associated with CM,^26^ suggesting that migraine chronification—and potentially refractory migraine—is caused by environmental rather than genetic factors.

The trigeminal system, including the peripheral innervation of the intracranial vasculature and meninges and its central input to the trigeminocervical complex (TCC), is important in migraine pathophysiology.^27^ From the TCC, sensory information is transmitted to the thalamus, sensory cortex, and multiple other cerebral areas that elaborate head pain. The hypothalamus is also implicated in migraine pathophysiology: hyperactivation of the anterior hypothalamus has been linked to migraine chronification due to hyperactivation of the limbic system.^28^ Interestingly, the hypothalamus hosts different neuropeptide systems, including orexins, oxytocin, neuropeptide Y, and pituitary adenylate cyclase activating protein (PACAP), which modulate neural function.^29^ We can speculate that altered connectivity between the hypothalamus and the limbic system contributes to the development of resistant and refractory migraine via those neuropeptides.

Central sensitisation, one of the driving factors of CM, lowers the threshold for trigeminal activation.^30^ Sustained neural activation events like central sensitisation might lead to the development of neuroplastic changes including structural remodelling of synaptic contacts on spinal dorsal horn neurons, reorganisation of cortical sensory maps, and increased activation of emotional networks in the brain.^31^ A MRI study revealed diffusion abnormalities in the thalamus, caudate, putamen, pallidum, amygdala, brainstem, and cerebral white matter of individuals with refractory migraine compared with headache-free controls.^32^ However, the study could not pinpoint specific alterations that are unique to refractory compared with non-resistant migraine. The activity of the amygdala, one of the key components of the limbic system, has been implicated in the development of nocebo responses^33^ which can be common in individuals with refractory migraine.^7^

No molecular biomarker is available to predict refractory migraine or the risk for it. CGRP levels are higher in individuals with CM compared to those with EM or healthy controls.^34^ However, CGRP is not the sole driver of disease worsening. Dopaminergic dysfunction might be a driver of migraine refractoriness, as suggested by the efficacy of olanzapine in individuals for whom CGRP-mAbs have failed.^35^ Increased glutamatergic transmission is also known to drive central sensitisation^36^ and could thus be a potential mechanism leading to resistance to treatments. Additionally, the endocannabinoid anti-nociceptive and anti-inflammatory system might be less functional in individuals with CM compared with those with EM.^37^ Other systems involved in migraine worsening include the kappa opioid receptors,^38^ the K_ATP_ receptors,^39^ and prolactin.^40^ Although those dysfunctions have not been specifically linked to resistance to treatments, they are linked to migraine worsening and could be potential pharmacological targets.

## Treatment

**Fig. 4:**
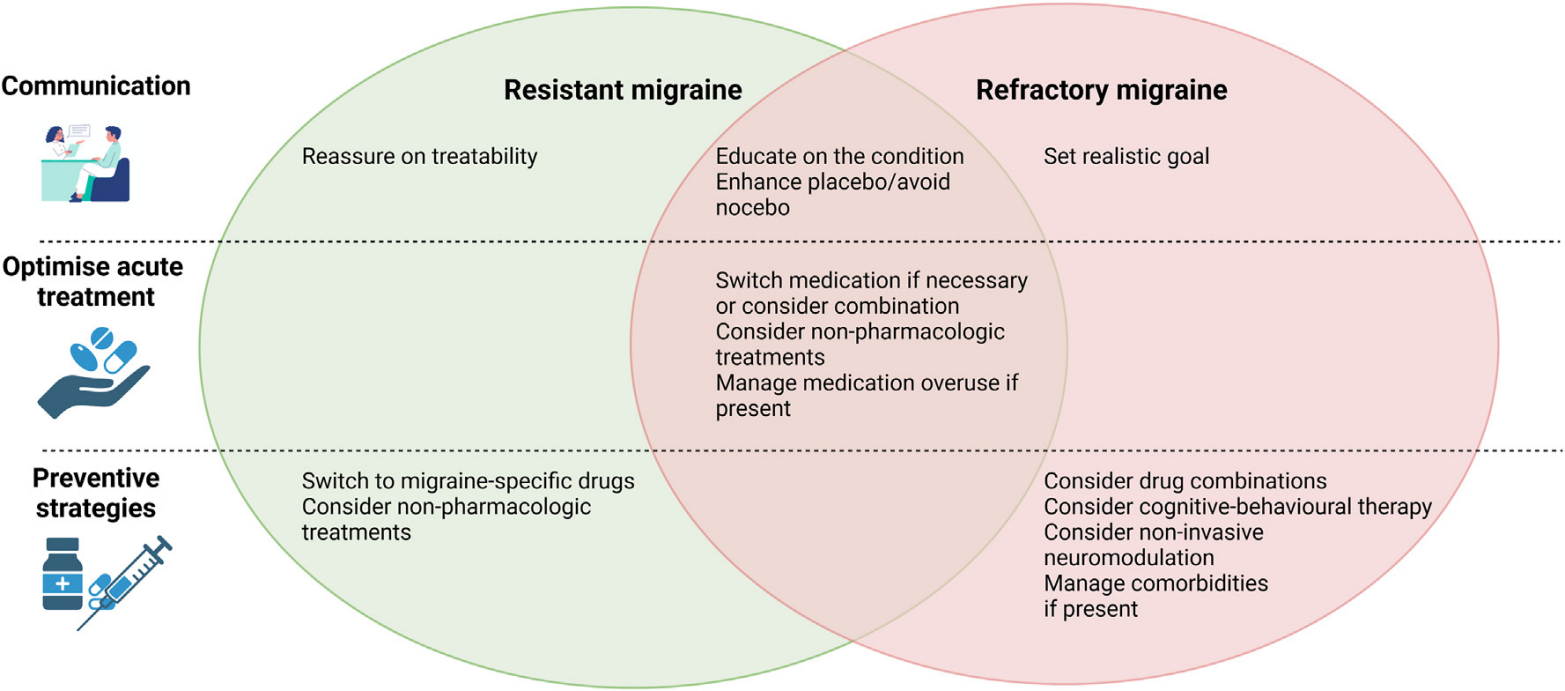
**Management of resistant and refractory migraine**. Approaches to resistant and refractory migraine, which includes a correct diagnosis, an adequate management of medication overuse and a proper choice of preventative strategies according to individuals' characteristics. The proposed approaches are not specific to either resistant or refractory migraine but represent priorities to each condition.

The treatment of resistant and refractory migraine requires careful clinical assessment of the individual, optimisation of acute and preventive treatment, management of comorbidities, and psychological support (Fig. 4).

An important step is proper communication. Individuals with resistant migraine should be reassured on the treatable nature of their condition, while those with refractory migraine should be provided with reasonable goals, taught to accept their diagnosis and to cope with the disease. Correct communication should also relieve the frustration of individuals experiencing multiple treatment failures and medical evaluations.

When assessing treatment failures, clinicians should assess the individuals’ attitudes towards preventive treatments together with their actual effects. Individuals with migraine might be dissatisfied about treatments and develop nocebo.^22^ They might be reluctant to take preventive treatments mostly developed for other indications and might underestimate the effectiveness of their treatments due to low expectations of efficacy. Possible reasons to contraindicate preventive treatments should also be carefully reviewed and not overestimated.

### Acute treatments and medication overuse

In resistant or refractory migraine, suboptimal response to acute treatments may increase the risk for medication overuse. Opioids have poor evidence of efficacy in migraine and are associated with a high risk of overuse.^41^ They therefore should be avoided in individuals with previous experience of failed treatments.^42^ Ditans (molecules targeting the 5-HT1F receptor inhibiting the release of CGRP from presynaptic neurons) and gepants are suitable for individuals not responding to triptans (5-HT1B/D receptor agonists).^43^ Animal evidence suggests that gepants present a low risk of medication overuse.^44^ However, we have no strong data on the risk of overuse of ditans and gepants in clinical practice as they are still to be widely employed.

When overuse occurs, withdrawal and detoxification should be considered. There are different detoxification protocols, all focused on withdrawal and education on the relevance and consequences of overuse.^45^ Psychiatric or physical comorbidities, poor psycho-social environment, relapse after a previous detoxification treatment, and daily use of acute treatments deserve attention as they are associated with failure of withdrawal schemes.^46^ Randomised controlled trials (RCTs) suggest combining acute medication withdrawal with preventive treatments.^47^^,^^48^

### Preventive treatment

Individuals with resistant and refractory migraine can be treated with pharmacological and/or non-pharmacological options. Pharmacological options can be used either as single treatments or in combination.

Non-pharmacological options should be part of the overall management of migraine but represent a pillar in the treatment of resistant and refractory migraine. Those interventions include behavioural therapies—including lifestyle adaptations–and neuromodulation.

A useful strategy is to encourage positive expectations on treatments that can modulate pain and analgesic treatment effects.^49^

#### Pharmacological options for resistant migraine

Individuals with resistant migraine with a failure of oral preventatives can be managed with onabotulinumtoxinA—for CM only—and drugs antagonising the CGRP pathway. The reason for intending these treatments as “second-line” is only based on their cost as there are no clinical or pharmacological reasons for distinguishing several lines of migraine treatments.

Although not specifically tested in RCTs, in clinical practice onabotulinumtoxinA has been reserved to individuals with CM and failure of oral drugs, in whom it proved high effectiveness and tolerability.^50^

RCTs of CGRP-mAbs were the first to prove the efficacy of migraine prevention in individuals with prior preventive treatment failures (Table 1).^51^, ^52^, ^53^, ^54^ Coupled with their high tolerability and adherence rate, CGRP-mAbs are a mainstay in the treatment of resistant migraine. Real-world studies confirmed CGRP-mAbs effectiveness and safety in individuals with many preventive treatment failures, mostly diagnosed with CM and medication overuse, and with severe migraine-related disability.^55^^,^^56^ In these studies, the proportion of individuals reporting a ≥50% reduction in MMDs from baseline—a common efficacy outcome in clinical studies–ranged between 30% and 51% at 3 months.^55^^,^^56^ No clear correlation exists between the number of prior preventative failures and response rate: a subgroup analysis of the FOCUS trial of fremanezumab reported a decrease in placebo response but not of drug efficacy among patients who had higher numbers of treatment failures^57^; conversely, real-world evidence suggests that a high number of preventive treatment failures might predict non-response to CGRP-mAbs.^58^^,^^59^

**Table 1:** Randomised controlled trials evaluating the 12-week efficacy of monoclonal antibodies acting on the CGRP pathway in patients with two to four prior preventative drug failures.

Study, year, NCT	Treatment, dose	Sample size, N^a^	Drop out due to lack of efficacy, N	Drop out due to side effects, N	Migraine form: EM and CM, %	Mean age ± SD	Female sex, %	MMDS difference from baseline, mean ± SD	≥50% response rate, %	≥75% response rate, %
CONQUER NCT03559257^51^	Galcanezumab LD 240 mg + 120 mg monthly	232	1	1	59; 41	45.9 (11.3)	84	−4.1 (0.3)	37.7∗	14.5∗
	Placebo	230	1	0	57; 43	45.7 (12.3)	88	−1 (0.3)	13.3∗	3.3∗
LIBERTY NCT03096834^52^	Erenumab 140 mg every 28 days	121	0	0	100	44.6 (10.5)	80	−1.8 (0.4)	30	12
	Placebo	125	0	0	100	44.2 (10.6)	82	−0.2 (0.4)	14	4
FOCUS NCT03308968^53^	Fremanezumab 225 mg monthly	283	0	4	39; 61	45.9 (11.1)	84	−3.7 (0.3)	34	N/A
	Fremanezumab 675 mg quarterly	276	1	1	39; 61	45.8 (11.0)	83	−4.1 (0.3)	34	N/A
	Placebo	279	1	3	40; 60	46.8 (11.1)	84	−0.6 (0.3)	9	N/A
DELIVER NCT04418765^54^	Eptinezumab 300 mg quarterly	293	0	6	54; 46	43.1 (10.2)	89	−5.3 (0.4)	49	19
	Eptinezumab 100 mg quarterly	299	3	1	54; 46	44.6 (10.8)	93	−4.8 (0.4)	42	16
	Placebo	298	1	1	55; 55	43.8 (10.8)	88	−2.1 (0.4)	13	2

*CM*-chronic migraine; *EM*-episodic migraine; *LD*-loading dose; *MMDs*-monthly migraine days; *N*-number; *N/A*-not available; *SD*-standard deviation; ∗marks mean percentage of patients with ≥50% response rate over the 12-week double blind phase.

a Total patients included in the studies including those who dropped out due to lack of efficacy or side effects.

Rimegepant and atogepant–oral drugs antagonising the CGRP pathway—proved superior over placebo in reducing MMDs and in ≥50% response rate in individuals with EM or CM^60^, ^61^, ^62^, ^63^ and up to four preventive treatment failures.

OnabotulinumtoxinA and drugs antagonising the CGRP pathway could help overcome tolerability issues associated with oral treatments and therefore increase adherence. Both CGRP-mAbs and onabotulinumtoxinA have a very low proportion of discontinuation for lack of efficacy or adverse events^50^, ^51^, ^52^, ^53^, ^54^^,^^64^ (Table 1). The HER-MES double-blind RCT proved the higher tolerability of and adherence to erenumab over topiramate.^65^ Gepants also did not lead to more adverse events than placebo in RCTs.^60^^,^^62^ The loss of treatment effect over time with oral agents might in theory be counteracted by dose increase, switch to another drug, or drug combination to regain therapeutic benefit, but the effectiveness of those strategies is not proven by literature data. Notably, the available data do not indicate a loss of treatment effect over time with drugs antagonising the CGRP pathway.^66^

#### Pharmacological options for refractory migraine

For individuals with refractory migraine, pharmacological options should be part of a multidisciplinary approach. Given the numerous treatment failures, clinicians should avoid the stress and negative expectations caused by continuing treatment attempts and focus on person-centred care. Combined pharmacological treatments might represent a possible strategy for individuals with a partial or inadequate response. Case series and retrospective studies showed possible efficacy of dual therapy with onabotulinumtoxinA and CGRP-mAbs in individuals who had not responded to all or most of the available treatments.^67^

#### Behavioural therapies

Behavioural therapies have not been specifically tested in resistant or refractory migraine. RCTs^68^^,^^69^ revealed that there is a synergistic, not just additive, benefit of a combination of pharmacologic treatment and behavioural therapy. Current use of behavioural therapies in patients with refractory migraine is limited by lack of evidence regarding efficacy and limited availability in headache centres, due to lack of personnel and resources.

#### Neuromodulation

Among neuromodulation techniques, occipital nerve stimulation (ONS), spinal cord stimulation (SCS), transcutaneous vagal nerve stimulation (tVNS), and single-pulse transcranial magnetic stimulation (sTMS) have been tested in individuals with refractory migraine.

ONS bases its rationale on the convergence of the greater occipital nerve on the same second order neurons within the TCC. RCTs proved the superiority of ONS to sham in 50% reduction of headache days per month, pain intensity,^70^ and disability^71^ in individuals with refractory CM, even if at the expense of adverse events, including minor infections and lead migration.

tVNS, which non-invasively targets the cervical branch of the vagus nerve using a small handheld device to stimulate the nerve in the region of the neck, is thought to activate low-threshold myelinated A-fibres, producing an antinociceptive effect on the second-order neurons of the spinothalamic and spino-reticular tracts within the TCC. However, the EVENT RCT did not show efficacy of the treatment in individuals with CM.^72^

sTMS applies single magnetic pulses inducing small electric currents in the occipital cortex. A real-world evidence clinical audit in the UK investigated daily sTMS application in individuals with at least three failures of established migraine preventive treatment over the course of 12 months. At 3 months, there was a median reduction of 5.0 monthly headache days compared to baseline (from 18.0 to 13.0 days), with 93/153 (60%) individuals achieving at least a 30% reduction in monthly migraine days. At 12 months, 69 (45%) of patients continued to have a sustained response to sTMS.^73^

SCS involves implanted electrical leads positioned epidurally at the C2 vertebral level. High-frequency paraesthesia-free stimulation (10 KHz) sends mild electrical pulses to the epidural space, which is thought to modulate nociceptive transmission at the level of second order neurons in the TCC. Real-world studies showed a long-term (>1 year) decrease in the number of MMDs and improved quality-of-life scores. About half of participants converted from CM to EM.^74^^,^^75^ Adverse events include lead migration and infections.

Taken together, neuromodulation approaches to refractory migraine reported mixed results in observational studies and small RCTs. It should be noted that studies of neuromodulation are small and including individuals in larger real-world studies and/or RCTs would be reasonable. Non-invasive techniques are widely applicable, while invasive techniques should be reserved to individuals with refractory migraine in the largest headache centres with multidisciplinary care and high-resource settings. Combinations between pharmacological and neuromodulation treatments could be considered in individuals with refractory migraine.

#### Other therapies

Other therapeutic approaches—both pharmacological and non-pharmacological—are used in migraine prevention and their use in individuals resistant to other treatments should be considered. Among pharmacological interventions, occipital or multiple cranial nerve blocks with local anaesthetics and/or steroids^76^ and intravenous infusions of anaesthetic agents such as lidocaine^77^ are often used to block severe migraine and facilitating long-term prevention. Among non-pharmacological approaches, physical therapy^78^ should be mentioned. Although not specifically tested in individuals with resistant or refractory migraine—and despite their overall low level of evidence—the use of those treatments could be considered in individuals with failure of all the other available treatments. For those poorly studied treatments, n-of-1 trials in which a single individual takes both the active drug or placebo in a sequential fashion could help providing preliminary efficacy data and developing new treatment protocols.

## Outstanding questions

The main outstanding questions for the understanding of resistant and refractory migraine refer to its identification, management, and pathogenesis.

Resistant and refractory migraine represent the progression of more manageable forms of migraine. Therefore, identifying the drivers of that progression is key to prevent their onset. Epidemiological studies specifically addressing the impact of resistant and refractory migraine and their trajectories over time are advisable in the future. The role of risk factors and triggers that can impact on resistant and refractory migraine also needs to be better understood. New tools are needed to better predict the development of resistant or refractory migraine at the individual level; artificial intelligence is being used to develop predictive models for that purpose.^79^

Regarding management, future research should focus on preventing treatment refractoriness. The use of specific migraine preventive agents in the early stages of progression might in theory prevent resistant or refractory forms. Solid evidence is still needed in the field; however, real-world data suggest that individuals with a shorter duration of CM respond better to onabotulinumtoxinA than those with longer disease duration.^80^ The early use of migraine-specific preventive treatments is limited due to reimbursement and accessibility issues; future policy changes may help tackle resistant and refractory migraine. A German study showed that erenumab use was more effective when reimbursed after only one prior preventive treatment failure than under stricter reimbursement conditions.^81^ At the same time, non-pharmacological approaches to migraine should be encouraged in an early phase of the disorder to prevent the onset of resistance to treatments; further RCTs on those approaches are warranted.

A further open question regarding the treatment of individuals with refractory migraine is the role of combined preventive treatments. The combination of treatments with peripheral actions—targeting the CGRP pathway—with those directly targeting the CNS might decrease both peripheral and central sensitisation and provide advantage in individuals with refractory forms. An alternative avenue for the development of new treatments is the improvement of established preventative drugs, including recombinant botulinum toxins^82^ and TMS stimulation protocols utilising theta burst stimulation.^83^ RCTs could also be specifically designed for individuals with resistant or refractory migraine and test higher doses or longer duration of current treatments.

Future research in the field of resistant and refractory migraine should also focus on contextual effects of treatments. The placebo effect is an issue in RCTs while it could be cultivated and enhanced in clinical practice as it increases the benefit of open-label treatments. On the other hand, nocebo effect should be avoided to enhance treatment adherence. Communication skills should be nurtured by clinicians addressing individuals with resistant and refractory migraine to help manage the placebo and nocebo effects.

Besides, the future definitions of resistant and refractory migraine should better consider failure of acute treatments. Efforts are ongoing to provide detailed definitions of failure to acute medication^43^ to be added to the definitions of resistant and refractory migraine. Notably, with the advent of gepants, CGRP inhibition is becoming the basis for both acute and preventive treatment of migraine and the same drug could be used for both purposes,^62^^,^^84^ possibly leading to changes in migraine treatment approaches.

Lastly, there is a need for improvement in pre-clinical and translational research on the pathogenesis of resistant and refractory migraine. Currently, preclinical models of migraine focus on the generation of attacks, but not on the factors impairing response to preventive treatments. The PACAP, dopaminergic, glutamatergic, and endocannabinoid systems may represent interesting targets for the development of future preventive drugs. New preventive drugs targeting pathophysiological mechanisms will likely reduce the number of individuals with refractory migraine.

## Conclusions

Treatment-related and individual factors might lead an individual with migraine to a resistant form, still treatable with specific drugs, or a refractory form, that cannot be managed satisfactorily with any treatment. Individuals with resistant or refractory migraine should be referred to expert headache care; in those with resistant migraine the preferential approach includes migraine-specific medications, while those with refractory migraine should receive a multidimensional approach in which pharmacological and non-pharmacological treatments are coupled with management of behavioural and psychological factors. In theory, managing migraine in its early years can prevent resistance and refractoriness. Development of personalised tools to predict refractoriness is a research priority that might finally lead to the development of targeted strategies changing the course of the disease.

## Contributors

SS conceived and structured the review and supervised the whole project. RO and EDM drafted the first manuscript. SS, APA, TPJ, and MTM contributed to parts of the manuscript and revised the first draft for intellectual content. All authors read and approved the final version of the manuscript.

## Declaration of interests

RO reports consulting fees from Teva, direct payments from Teva, Eli Lilly, Novartis, AbbVie, and Pfizer, support for attending meetings and/or travel from Novartis and Teva, participation to advisory boards from Eli Lilly, and other support from Novartis and Allergan-AbbVie; he is a Junior Editorial Board member of The Journal of Headache and Pain. SS reports grants from Novartis and Uriach, consulting fees from Abbott, Allergan, AbbVie, Novartis, Teva, Eli Lilly, Pfizer, Lundbeck, Novo Nordisk, and AstraZeneca, direct payment from Abbott, Allergan-AbbVie, Novartis, Teva, Eli Lilly, Pfizer, Lundbeck, Novo Nordisk, and AstraZeneca, support for attending meetings and/or travel from Eli Lilly, Novartis, Teva, and Lundbeck, receipt of equipment from Allergan-AbbVie and Novo Nordisk; she is President Elect of the European Stroke Organisation and Editor-in-Chief of Cephalalgia. APA received grants or contracts from Brain Research UK, Medical Research Council, Medical Research Foundation, Rosetrees, and Migraine Trust, consulting fees from Eli Lilly and AbbVie, direct payment from Eli Lilly, AbbVie, eNeura, Autonomic Technologies, and Novartis, participation to advisory boards from Eli Lilly and AbbVie; she is Chair of the Communications Committee of the International Headache Society and Chair of the Headache SIG of the British Pain Society. TPJ reports grants from the European Regional Development Funds (ERDF), Innovation Fund of the Federal Joint Committee (Germany) and Novartis; compensation from Allergan, Abbvie, Chordate, Grünenthal, Hormosan, Lilly, Lundbeck, Novartis, Pfizer, Teva and Sanofi for consultant services and/or speaker honoraria; he is President of the German Migraine and Headache Society and member of the Educational Committee of the German Pain Society. EDM and MTM report no conflict of interest.
